# (2*E*)-3-(4-Bromo­phen­yl)-1-(2-methyl-4-phenyl-3-quinol­yl)prop-2-en-1-one

**DOI:** 10.1107/S1600536810013784

**Published:** 2010-04-17

**Authors:** R. Prasath, S. Sarveswari, V. Vijayakumar, T. Narasimhamurthy, Edward R. T. Tiekink

**Affiliations:** a Organic Chemistry Division, School of Advanced Sciences, VIT University, Vellore 632 014, India; b Materials Research Centre, Indian Institute of Science, Bengaluru 560 012, India; c Department of Chemistry, University of Malaya, 50603 Kuala Lumpur, Malaysia

## Abstract

The conformation about the ethene bond [1.316 (3) Å] in the title compound, C_25_H_18_BrNO, is *E*. The quinoline ring forms dihedral angles of 67.21 (10) and 71.68 (10)° with the benzene and bromo-substituted benzene rings, respectively. Highlighting the non-planar arrangement of aromatic rings, the dihedral angle formed between the benzene rings is 58.57 (12)°.

## Related literature

For general background to quinoline derivatives, see: Morimoto *et al.* (1991 ▶); Michael (1997 ▶); Markees *et al.* (1970 ▶); Campbell *et al.* (1998 ▶); Maguire *et al.* (1994 ▶); Kalluraya & Sreenivasa (1998 ▶); Roma *et al.* (2000 ▶); Chen *et al.* (2001 ▶). For inter­est in the biological activities of chalcones, see: Dimmock *et al.* (1999 ▶).
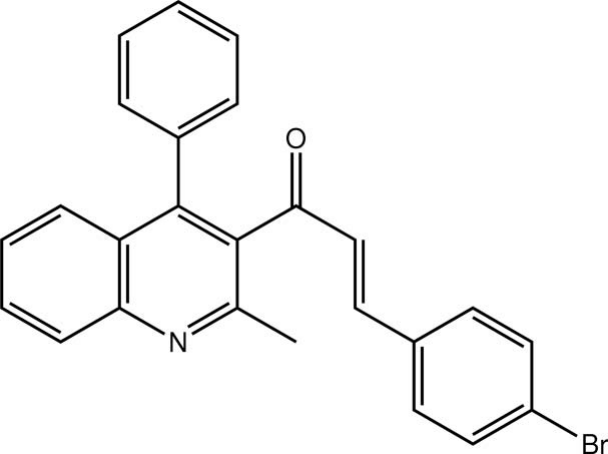


## Experimental

### 

#### Crystal data

C_25_H_18_BrNO
*M* *_r_* = 428.31
Triclinic, 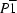

*a* = 6.6407 (3) Å
*b* = 10.0395 (4) Å
*c* = 15.5193 (6) Å
α = 92.192 (2)°
β = 95.234 (2)°
γ = 105.869 (2)°
*V* = 988.92 (7) Å^3^
*Z* = 2
Mo *K*α radiation
μ = 2.09 mm^−1^
*T* = 293 K
0.28 × 0.21 × 0.14 mm

#### Data collection

Bruker SMART APEX CCD diffractometer
Absorption correction: multi-scan (*SADABS*; Bruker, 1998 ▶) *T*_min_ = 0.596, *T*_max_ = 0.746
15973 measured reflections
3470 independent reflections
2545 reflections with *I* > 2σ(*I*)
*R*_int_ = 0.027

#### Refinement

*R*[*F* ^2^ > 2σ(*F* ^2^)] = 0.033
*wR*(*F* ^2^) = 0.083
*S* = 1.01
3470 reflections
254 parameters
H-atom parameters constrained
Δρ_max_ = 0.34 e Å^−3^
Δρ_min_ = −0.35 e Å^−3^

### 

Data collection: *SMART* (Bruker, 2001 ▶); cell refinement: *SAINT* (Bruker, 2001 ▶); data reduction: *SAINT*; program(s) used to solve structure: *SHELXS97* (Sheldrick, 2008 ▶); program(s) used to refine structure: *SHELXL97* (Sheldrick, 2008 ▶) and *PLATON* (Spek, 2009 ▶); molecular graphics: *ORTEP-3* (Farrugia, 1997 ▶); software used to prepare material for publication: *publCIF* (Westrip, 2010 ▶).

## Supplementary Material

Crystal structure: contains datablocks global, I. DOI: 10.1107/S1600536810013784/hg2673sup1.cif

Structure factors: contains datablocks I. DOI: 10.1107/S1600536810013784/hg2673Isup2.hkl

Additional supplementary materials:  crystallographic information; 3D view; checkCIF report

## Figures and Tables

**Fig. 1.**
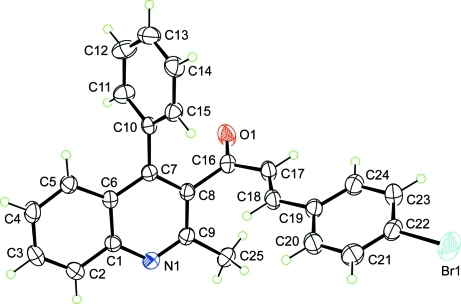
The molecular structure of (I) showing the atom-labelling scheme and displacement ellipsoids at the 35% probability level.

## supplementary crystallographic information

### Comment

Natural products (Morimoto *et al.*, 1991; Michael, 1997) and biologically active compounds (Markees *et al.*, 1970; Campbell *et al.*, 1998) are known to contain the quinoline nucleus. Quinolines are also known to possess attractive applications as pharmaceuticals and agrochemicals (Maguire *et al.*, 1994; Kalluraya & Sreenivasa, 1998; Roma *et al.*, 2000; Chen *et al.*, 2001). The open chain flavanoids, the chalcones, also possess a variety of biological activities (Dimmock *et al.*, 1999). Herein, we report the synthesis and crystal structure of a molecule containing both quinoline and chalcone groups, (I).

In the structure of (I), the conformation about the C17═C18 [1.316 (3) Å] bond is *E*, Fig. 1. The chalcone residue is essentially planar as seen in the O1–C16–C17–C18 torsion angle of 173.7 (2) °. While the planarity extends out to the 4-bromobenzene ring [the C17–C18–C19–C20 torsion angle is -175.6 (2) °] this is not true for the quinoline residue (r.m.s. deviation = 0.0164 Å) which is twisted out of the plane through the chalcone residue: the C7–C8–C16–C17 torsion angle is 112.9 (2) °. In the same way, the C7-bound benzene ring is significantly twisted out of the plane of the quinoline ring as seen in the C6–C7–C10–C11 torsion angle of -67.0 (3) °. The non-planar nature of the molecule is reflected in the dihedral angles formed between the quinoline molecule and the benzene and bromo-substituted benzene rings of 67.21 (10) and 71.68 (10) °, respectively; the dihedral angle formed between the benzene rings is 58.57 (12) °.

Except for some rather weak π···π interactions [ring centroid(N1,C1,C6–C9)···ring centroid(C1–C6)^i^ distance = 3.8124 (13) Å for *i*: 2-*x*, 2-*y*, -*z*] between centrosymmetrically related quinoline rings, no specific intermolecular forces are evident in the crystal packing.

### Experimental

A mixture of 3-acetyl-2-methyl-4-phenylquinoline (2.6 g 0.01 M) and 4-bromobenzaldehyde (1.84 g 0.01 M), and a catalytic amount of KOH in distilled ethanol was stirred for about 12 h. The resulting mixture was concentrated to remove ethanol, poured onto ice, and neutralized with dilute acetic acid. The resultant solid was filtered, dried, purified by column chromatography using 1:1 mixture of ethyl acetate and petroleum ether, and recrystallized using ethyl acetate; yield: 65 % and m.pt: 459 K.

### Refinement

The C-bound H atoms were geometrically placed (C–H = 0.93–0.96 Å) and refined as riding with *U_iso_*(H) = 1.2–1.5*U_eq_*(C).

### Crystal data

**Table:**

C_25_H_18_BrNO	*Z* = 2
*M**_r_* = 428.31	*F*(000) = 436
Triclinic, *P*1	*D*_x_ = 1.438 Mg m^−^^3^
Hall symbol: -P 1	Mo *K*α radiation, λ = 0.71073 Å
*a* = 6.6407 (3) Å	Cell parameters from 4795 reflections
*b* = 10.0395 (4) Å	θ = 2.6–23.7°
*c* = 15.5193 (6) Å	µ = 2.09 mm^−^^1^
α = 92.192 (2)°	*T* = 293 K
β = 95.234 (2)°	Plate, colourless
γ = 105.869 (2)°	0.28 × 0.21 × 0.14 mm
*V* = 988.92 (7) Å^3^	

### Data collection

**Table:**

Bruker SMART APEX CCD diffractometer	3470 independent reflections
Radiation source: fine-focus sealed tube	2545 reflections with *I* > 2σ(*I*)
graphite	*R*_int_ = 0.027
ω scans	θ_max_ = 25.0°, θ_min_ = 2.1°
Absorption correction: multi-scan (*SADABS*; Bruker, 1998)	*h* = −7→7
*T*_min_ = 0.596, *T*_max_ = 0.746	*k* = −11→11
15973 measured reflections	*l* = −18→18

### Refinement

**Table:**

Refinement on *F*^2^	Primary atom site location: structure-invariant direct methods
Least-squares matrix: full	Secondary atom site location: difference Fourier map
*R*[*F*^2^ > 2σ(*F*^2^)] = 0.033	Hydrogen site location: inferred from neighbouring sites
*wR*(*F*^2^) = 0.083	H-atom parameters constrained
*S* = 1.01	*w* = 1/[σ^2^(*F*_o_^2^) + (0.0378*P*)^2^ + 0.3122*P*] where *P* = (*F*_o_^2^ + 2*F*_c_^2^)/3
3470 reflections	(Δ/σ)_max_ = 0.001
254 parameters	Δρ_max_ = 0.34 e Å^−^^3^
0 restraints	Δρ_min_ = −0.35 e Å^−^^3^

### Special details

**Table:**

Geometry. All s.u.'s (except the s.u. in the dihedral angle between two l.s. planes) are estimated using the full covariance matrix. The cell s.u.'s are taken into account individually in the estimation of s.u.'s in distances, angles and torsion angles; correlations between s.u.'s in cell parameters are only used when they are defined by crystal symmetry. An approximate (isotropic) treatment of cell s.u.'s is used for estimating s.u.'s involving l.s. planes.
Refinement. Refinement of *F*^2^ against ALL reflections. The weighted *R*-factor wR and goodness of fit *S* are based on *F*^2^, conventional *R*-factors *R* are based on *F*, with *F* set to zero for negative *F*^2^. The threshold expression of *F*^2^ > 2σ(*F*^2^) is used only for calculating *R*-factors(gt) etc. and is not relevant to the choice of reflections for refinement. *R*-factors based on *F*^2^ are statistically about twice as large as those based on *F*, and *R*- factors based on ALL data will be even larger.

### Fractional atomic coordinates and isotropic or equivalent isotropic displacement parameters (Å^2^)

**Table:**

	*x*	*y*	*z*	*U*_iso_*/*U*_eq_	
Br1	0.08613 (5)	0.24943 (4)	0.49005 (2)	0.08720 (16)	
O1	1.1467 (3)	0.65490 (17)	0.15079 (12)	0.0608 (5)	
N1	0.7271 (3)	0.91386 (18)	0.04962 (12)	0.0419 (4)	
C1	0.8532 (3)	1.0419 (2)	0.07979 (14)	0.0377 (5)	
C2	0.8037 (4)	1.1593 (2)	0.04646 (15)	0.0475 (6)	
H2	0.6909	1.1479	0.0040	0.057*	
C3	0.9207 (4)	1.2891 (2)	0.07632 (17)	0.0540 (6)	
H3	0.8868	1.3658	0.0541	0.065*	
C4	1.0909 (4)	1.3087 (2)	0.13987 (16)	0.0527 (6)	
H4	1.1685	1.3982	0.1598	0.063*	
C5	1.1440 (4)	1.1977 (2)	0.17279 (14)	0.0431 (5)	
H5	1.2583	1.2119	0.2148	0.052*	
C6	1.0268 (3)	1.0609 (2)	0.14360 (13)	0.0348 (5)	
C7	1.0703 (3)	0.9398 (2)	0.17656 (13)	0.0344 (5)	
C8	0.9391 (3)	0.8119 (2)	0.14548 (14)	0.0361 (5)	
C9	0.7675 (3)	0.8033 (2)	0.08138 (14)	0.0395 (5)	
C10	1.2501 (3)	0.9522 (2)	0.24382 (13)	0.0361 (5)	
C11	1.4566 (4)	1.0046 (3)	0.22479 (16)	0.0522 (6)	
H11	1.4833	1.0335	0.1697	0.063*	
C12	1.6221 (4)	1.0137 (3)	0.28757 (19)	0.0627 (7)	
H12	1.7597	1.0486	0.2743	0.075*	
C13	1.5861 (4)	0.9721 (3)	0.36906 (18)	0.0591 (7)	
H13	1.6984	0.9783	0.4109	0.071*	
C14	1.3823 (4)	0.9209 (3)	0.38841 (16)	0.0563 (6)	
H14	1.3568	0.8921	0.4436	0.068*	
C15	1.2162 (4)	0.9120 (2)	0.32667 (14)	0.0451 (5)	
H15	1.0791	0.8784	0.3409	0.054*	
C16	0.9910 (4)	0.6815 (2)	0.17362 (15)	0.0433 (5)	
C17	0.8555 (4)	0.5880 (2)	0.22805 (16)	0.0488 (6)	
H17	0.8854	0.5047	0.2391	0.059*	
C18	0.6945 (4)	0.6130 (2)	0.26255 (15)	0.0457 (6)	
H18	0.6660	0.6964	0.2503	0.055*	
C19	0.5548 (3)	0.5238 (2)	0.31818 (15)	0.0444 (5)	
C20	0.3842 (4)	0.5633 (3)	0.34362 (17)	0.0547 (6)	
H20	0.3621	0.6461	0.3258	0.066*	
C21	0.2463 (4)	0.4832 (3)	0.39470 (17)	0.0602 (7)	
H21	0.1321	0.5112	0.4110	0.072*	
C22	0.2791 (4)	0.3614 (3)	0.42126 (16)	0.0524 (6)	
C23	0.4469 (4)	0.3192 (3)	0.39775 (17)	0.0580 (7)	
H23	0.4680	0.2365	0.4161	0.070*	
C24	0.5839 (4)	0.4003 (2)	0.34676 (17)	0.0550 (6)	
H24	0.6984	0.3719	0.3311	0.066*	
C26	0.6217 (4)	0.6658 (2)	0.04495 (17)	0.0538 (6)	
H26A	0.5103	0.6364	0.0813	0.081*	
H26B	0.6990	0.5979	0.0430	0.081*	
H26C	0.5630	0.6755	−0.0126	0.081*	

### Atomic displacement parameters (Å^2^)

**Table:**

	*U*^11^	*U*^22^	*U*^33^	*U*^12^	*U*^13^	*U*^23^
Br1	0.0736 (2)	0.0933 (3)	0.0832 (2)	−0.00470 (17)	0.02395 (17)	0.03235 (18)
O1	0.0616 (11)	0.0476 (10)	0.0839 (13)	0.0264 (8)	0.0242 (10)	0.0157 (9)
N1	0.0385 (10)	0.0394 (11)	0.0463 (11)	0.0092 (8)	0.0020 (8)	0.0015 (9)
C1	0.0377 (11)	0.0369 (13)	0.0403 (12)	0.0122 (10)	0.0072 (10)	0.0035 (10)
C2	0.0487 (13)	0.0461 (15)	0.0498 (14)	0.0180 (11)	−0.0005 (11)	0.0081 (11)
C3	0.0643 (16)	0.0394 (14)	0.0637 (16)	0.0230 (12)	0.0047 (13)	0.0121 (12)
C4	0.0617 (15)	0.0318 (13)	0.0623 (16)	0.0101 (11)	0.0052 (13)	−0.0014 (11)
C5	0.0463 (12)	0.0377 (13)	0.0433 (13)	0.0099 (10)	0.0007 (10)	0.0001 (10)
C6	0.0382 (11)	0.0316 (12)	0.0363 (12)	0.0102 (9)	0.0106 (9)	0.0029 (9)
C7	0.0337 (11)	0.0359 (12)	0.0355 (11)	0.0104 (9)	0.0099 (9)	0.0041 (9)
C8	0.0365 (11)	0.0323 (12)	0.0402 (12)	0.0088 (9)	0.0095 (10)	0.0049 (9)
C9	0.0383 (11)	0.0347 (12)	0.0446 (13)	0.0079 (9)	0.0080 (10)	0.0004 (10)
C10	0.0407 (12)	0.0288 (11)	0.0399 (12)	0.0116 (9)	0.0046 (10)	0.0006 (9)
C11	0.0430 (13)	0.0664 (16)	0.0482 (14)	0.0145 (12)	0.0112 (12)	0.0039 (12)
C12	0.0369 (13)	0.0794 (19)	0.0726 (19)	0.0188 (13)	0.0062 (13)	−0.0074 (15)
C13	0.0556 (16)	0.0624 (17)	0.0600 (18)	0.0249 (13)	−0.0123 (13)	−0.0056 (13)
C14	0.0676 (17)	0.0538 (15)	0.0437 (14)	0.0131 (13)	−0.0052 (13)	0.0080 (11)
C15	0.0448 (13)	0.0447 (13)	0.0422 (13)	0.0059 (10)	0.0050 (11)	0.0061 (10)
C16	0.0461 (13)	0.0340 (12)	0.0482 (14)	0.0087 (10)	0.0048 (11)	0.0029 (10)
C17	0.0563 (14)	0.0332 (13)	0.0579 (15)	0.0122 (11)	0.0097 (12)	0.0089 (11)
C18	0.0529 (14)	0.0315 (12)	0.0524 (14)	0.0111 (10)	0.0051 (12)	0.0043 (10)
C19	0.0467 (13)	0.0358 (13)	0.0469 (14)	0.0058 (10)	0.0025 (11)	0.0035 (10)
C20	0.0575 (15)	0.0472 (14)	0.0639 (16)	0.0184 (12)	0.0138 (13)	0.0121 (12)
C21	0.0506 (15)	0.0665 (18)	0.0658 (17)	0.0163 (13)	0.0162 (13)	0.0077 (14)
C22	0.0493 (14)	0.0513 (15)	0.0471 (14)	−0.0020 (12)	0.0036 (11)	0.0073 (11)
C23	0.0620 (16)	0.0439 (14)	0.0653 (17)	0.0088 (12)	0.0061 (13)	0.0146 (12)
C24	0.0489 (14)	0.0479 (15)	0.0687 (17)	0.0117 (11)	0.0122 (13)	0.0087 (13)
C26	0.0485 (13)	0.0431 (14)	0.0615 (16)	0.0026 (11)	−0.0037 (12)	−0.0033 (12)

### Geometric parameters (Å, °)

**Table:**

Br1—C22	1.897 (2)	C12—H12	0.9300
O1—C16	1.215 (3)	C13—C14	1.375 (4)
N1—C9	1.314 (3)	C13—H13	0.9300
N1—C1	1.367 (3)	C14—C15	1.375 (3)
C1—C2	1.411 (3)	C14—H14	0.9300
C1—C6	1.415 (3)	C15—H15	0.9300
C2—C3	1.360 (3)	C16—C17	1.463 (3)
C2—H2	0.9300	C17—C18	1.316 (3)
C3—C4	1.396 (3)	C17—H17	0.9300
C3—H3	0.9300	C18—C19	1.469 (3)
C4—C5	1.361 (3)	C18—H18	0.9300
C4—H4	0.9300	C19—C20	1.383 (3)
C5—C6	1.415 (3)	C19—C24	1.389 (3)
C5—H5	0.9300	C20—C21	1.376 (3)
C6—C7	1.427 (3)	C20—H20	0.9300
C7—C8	1.380 (3)	C21—C22	1.371 (4)
C7—C10	1.487 (3)	C21—H21	0.9300
C8—C9	1.424 (3)	C22—C23	1.369 (4)
C8—C16	1.513 (3)	C23—C24	1.376 (3)
C9—C26	1.507 (3)	C23—H23	0.9300
C10—C15	1.382 (3)	C24—H24	0.9300
C10—C11	1.392 (3)	C26—H26A	0.9600
C11—C12	1.381 (3)	C26—H26B	0.9600
C11—H11	0.9300	C26—H26C	0.9600
C12—C13	1.368 (4)		
			
C9—N1—C1	118.80 (18)	C15—C14—C13	120.4 (2)
N1—C1—C2	117.86 (19)	C15—C14—H14	119.8
N1—C1—C6	122.80 (18)	C13—C14—H14	119.8
C2—C1—C6	119.33 (19)	C14—C15—C10	120.9 (2)
C3—C2—C1	120.1 (2)	C14—C15—H15	119.6
C3—C2—H2	120.0	C10—C15—H15	119.6
C1—C2—H2	120.0	O1—C16—C17	120.1 (2)
C2—C3—C4	121.0 (2)	O1—C16—C8	119.41 (19)
C2—C3—H3	119.5	C17—C16—C8	120.4 (2)
C4—C3—H3	119.5	C18—C17—C16	125.0 (2)
C5—C4—C3	120.4 (2)	C18—C17—H17	117.5
C5—C4—H4	119.8	C16—C17—H17	117.5
C3—C4—H4	119.8	C17—C18—C19	127.3 (2)
C4—C5—C6	120.5 (2)	C17—C18—H18	116.4
C4—C5—H5	119.7	C19—C18—H18	116.4
C6—C5—H5	119.7	C20—C19—C24	117.6 (2)
C5—C6—C1	118.67 (18)	C20—C19—C18	119.0 (2)
C5—C6—C7	123.60 (19)	C24—C19—C18	123.4 (2)
C1—C6—C7	117.71 (18)	C21—C20—C19	121.6 (2)
C8—C7—C6	118.19 (19)	C21—C20—H20	119.2
C8—C7—C10	121.31 (18)	C19—C20—H20	119.2
C6—C7—C10	120.49 (18)	C20—C21—C22	119.2 (2)
C7—C8—C9	120.04 (18)	C20—C21—H21	120.4
C7—C8—C16	119.47 (18)	C22—C21—H21	120.4
C9—C8—C16	120.24 (18)	C23—C22—C21	120.9 (2)
N1—C9—C8	122.44 (19)	C23—C22—Br1	119.93 (19)
N1—C9—C26	115.76 (19)	C21—C22—Br1	119.18 (19)
C8—C9—C26	121.80 (19)	C22—C23—C24	119.4 (2)
C15—C10—C11	118.4 (2)	C22—C23—H23	120.3
C15—C10—C7	120.84 (19)	C24—C23—H23	120.3
C11—C10—C7	120.77 (19)	C23—C24—C19	121.3 (2)
C12—C11—C10	120.1 (2)	C23—C24—H24	119.3
C12—C11—H11	119.9	C19—C24—H24	119.3
C10—C11—H11	119.9	C9—C26—H26A	109.5
C13—C12—C11	120.8 (2)	C9—C26—H26B	109.5
C13—C12—H12	119.6	H26A—C26—H26B	109.5
C11—C12—H12	119.6	C9—C26—H26C	109.5
C12—C13—C14	119.3 (2)	H26A—C26—H26C	109.5
C12—C13—H13	120.3	H26B—C26—H26C	109.5
C14—C13—H13	120.3		
			
C9—N1—C1—C2	−178.4 (2)	C8—C7—C10—C11	114.2 (2)
C9—N1—C1—C6	0.4 (3)	C6—C7—C10—C11	−67.0 (3)
N1—C1—C2—C3	178.0 (2)	C15—C10—C11—C12	0.8 (3)
C6—C1—C2—C3	−0.7 (3)	C7—C10—C11—C12	−179.1 (2)
C1—C2—C3—C4	0.1 (4)	C10—C11—C12—C13	−0.2 (4)
C2—C3—C4—C5	0.5 (4)	C11—C12—C13—C14	−0.2 (4)
C3—C4—C5—C6	−0.5 (4)	C12—C13—C14—C15	−0.2 (4)
C4—C5—C6—C1	−0.2 (3)	C13—C14—C15—C10	0.9 (4)
C4—C5—C6—C7	−178.5 (2)	C11—C10—C15—C14	−1.2 (3)
N1—C1—C6—C5	−177.97 (19)	C7—C10—C15—C14	178.7 (2)
C2—C1—C6—C5	0.8 (3)	C7—C8—C16—O1	−66.9 (3)
N1—C1—C6—C7	0.5 (3)	C9—C8—C16—O1	107.3 (2)
C2—C1—C6—C7	179.20 (19)	C7—C8—C16—C17	112.9 (2)
C5—C6—C7—C8	177.32 (19)	C9—C8—C16—C17	−72.8 (3)
C1—C6—C7—C8	−1.0 (3)	O1—C16—C17—C18	173.7 (2)
C5—C6—C7—C10	−1.5 (3)	C8—C16—C17—C18	−6.1 (4)
C1—C6—C7—C10	−179.90 (18)	C16—C17—C18—C19	−179.4 (2)
C6—C7—C8—C9	0.8 (3)	C17—C18—C19—C20	−175.6 (2)
C10—C7—C8—C9	179.66 (18)	C17—C18—C19—C24	4.1 (4)
C6—C7—C8—C16	175.09 (18)	C24—C19—C20—C21	−0.6 (4)
C10—C7—C8—C16	−6.1 (3)	C18—C19—C20—C21	179.1 (2)
C1—N1—C9—C8	−0.6 (3)	C19—C20—C21—C22	0.2 (4)
C1—N1—C9—C26	−179.99 (19)	C20—C21—C22—C23	0.1 (4)
C7—C8—C9—N1	0.0 (3)	C20—C21—C22—Br1	−178.94 (19)
C16—C8—C9—N1	−174.19 (19)	C21—C22—C23—C24	0.0 (4)
C7—C8—C9—C26	179.3 (2)	Br1—C22—C23—C24	179.02 (19)
C16—C8—C9—C26	5.1 (3)	C22—C23—C24—C19	−0.4 (4)
C8—C7—C10—C15	−65.7 (3)	C20—C19—C24—C23	0.7 (4)
C6—C7—C10—C15	113.1 (2)	C18—C19—C24—C23	−179.0 (2)
